# Everolimus versus sirolimus for angiomyolipoma associated with tuberous sclerosis complex: a multi-institutional retrospective study in China

**DOI:** 10.1186/s13023-021-01932-z

**Published:** 2021-07-03

**Authors:** Cong Luo, Yu-Shi Zhang, Ming-Xin Zhang, Min-Feng Chen, Yuan Li, Lin Qi, Han-Zhong Li, Xiong-Bin Zu, Yi Cai

**Affiliations:** 1 Department of Urology, Xiangya Hospital, Central South University, No. 87 Xiangya Road, Changsha, 410008 Hunan People’s Republic of China; 2 National Clinical Research Center for Geriatric Disorders, Xiangya Hospital, Central South University, Changsha, 410008 Hunan People’s Republic of China; 3 Department of Urology, Peking Union Medical College Hospital, Chinese Academy of Medical Sciences and Peking Union Medical College, No.1 Shuaifuyuan, Dongcheng District, Beijing, 100730 People’s Republic of China; 4 Department of Urology, The Affiliated Hospital of Qingdao University, No. 16 Jiangsu Road, Shinan District, Qingdao, 266000 Shandong People’s Republic of China

**Keywords:** Tuberous sclerosis complex, Everolimus, Sirolimus, Treatment outcome, Adverse events

## Abstract

**Purpose:**

To evaluate the efficacy and safety of everolimus and sirolimus in patients with tuberous sclerosis complex-associated angiomyolipomas (TSC-AML).

**Materials and methods:**

We performed a multi-institutional retrospective study of TSC-AML patients treated with oral everolimus 10 mg or sirolimus 2 mg per day for at least 3 months. Angiomyolipoma volume was estimated using orthogonal measurements by MRI or CT. Adverse events (AEs) were assessed according to the National Cancer Institute Common Terminology Criteria for Adverse Events. All analyses were performed using SPSS 19.0 software.

**Results:**

Response rates were high in both groups. With the prolonged medication durations, the therapeutic efficacy of both agents became more significant. The TSC-AML volume reduction after 6 and 12 months was more pronounced in patients with everolimus than those with sirolimus. More than half of the patients treated with everolimus had ≥ 50% reduction, and approximately 80% of them had ≥ 30% reduction, which was higher than that in patients treated with sirolimus. Regarding safety, there was no significant difference in the incidence of AEs between the two groups.

**Conclusions:**

Both everolimus and sirolimus are excellent therapeutic options for TSC-AML. However, everolimus has a better therapeutic efficacy than sirolimus, particularly in reducing TSC-AML volume. Everolimus is therefore recommended as the first choice of therapy for TSC-AML.

## Introduction

Tuberous sclerosis complex (TSC) is a hereditary disorder with an incidence of 1:6,000–1:10,000. It is characterized by prominent neurodevelopmental features and hamartomas in multiple organs in the body, including brain, heart, lungs, bones, and especially kidneys [1]. Among the renal phenotypes of TSC, renal angiomyolipoma (AML) is the most common type affecting up to 80% of patients with TSC, which can lead to chronic kidney disease (CKD) and intrarenal hemorrhage [2]. Considering that renal disease is a leading cause of death or disability, second only to neurologic disease in the overall populations [3], clinicians have paid more attention to the renal involvement in TSC and actively seek more optimal treatment strategies.

TSC is an autosomal dominant disorder caused by mutations in either TSC1 or TSC2 gene. TSC1 and TSC2, together with a third subunit TBC1D7, form a functional complex and act as important repressors upstream of the mammalian target of rapamycin (mTOR). TSC1/TSC2 mutations will cause dysfunction of the complex, resulting in activation of the mTOR signaling pathway, thus inducing the occurrence of TSC-related benign tumors or hamartomas in multiple systems [4]. Based on this pathogenic mechanism, mTOR inhibitors have been considered as a novel therapy for TSC. Previous cohort studies have corroborated the therapeutic effect of mTOR inhibitors against TSC related complications, including subependymal giant cell astrocytomas, renal AML, seizure, and facial angiofibromas [5–8].

Sirolimus, a kind of mTOR inhibitor, was first isolated from Streptomyces hygroscopicus in a soil sample from Easter Island in 1975 [9]. Everolimus (RAD001) is derived from sirolimus, also known as 42-*O*-(2-Hydroxyethyl)-rapamycin. They differ mainly in pharmacokinetic characteristics. Everolimus demonstrates better absorption, higher oral bioavailability, faster steady-state blood concentration after administration, and faster elimination after withdrawal [10–12]. Besides, certain study showed that everolimus could preserve renal function in most patients [13]. However, the high economic costs and strict indications limit the accessibility and utilization of everolimus in Chinese mainland, as the price of everolimus is currently about ¥ 8800 per month, even up to ¥ 15,000 per month before being included in medical insurance. While the price of sirolimus is only ¥ 3200 per month, or even ¥ 1000 per month for domestic ones. And the indication for the use of sirolimus is not such strict.

Clinical studies have suggested that TSC-AML (≥ 3 cm in diameter) could respond to sirolimus or everolimus therapy [14, 15]. And we have also confirmed that TSC-AML regressed somewhat during everolimus therapy in a Chinese cohort [16]. However, there are no studies comparing outcomes of TSC patients undergoing everolimus or sirolimus treatment. Herein, we reported a multi-institutional retrospective study in China comparing the efficacy and safety of everolimus and sirolimus in TSC-AML patients.

## Methods

### Study design and patient population

This was a three center retrospective cohort study, including Xiangya Hospital of Central South University, Peking Union Medical College Hospital and The Affiliated Hospital of Qingdao University. It was carried out in accordance with the *Declaration of Helsinki *[17]. All patients signed written informed consent to voluntarily participate in this cohort. Inclusion criteria were: (1) ≥ 18 years of age; (2) presented between September 2014 and September 2020 to the department of urology; (3) with a definite diagnosis of TSC. Diagnosis was defined as including 2 major criteria or including 1 major criterion and ≥ 2 minor criteria, recommended by International Consensus Conference in 2012 TOSCA [18]; (4) with at least one AML with diameter ≥ 30 mm; (5) treated with oral everolimus or sirolimus monotherapy.

We enrolled patients who received oral everolimus 10 mg or sirolimus 2 mg per day for at least 3 months and got radiographic follow-up and safety evaluation at 3, 6, 12, 24 months. The primary efficacy endpoint was the proportion of patients with confirmed AML response of at least a 50% reduction in total volume of target AML relative to baseline.

### Treatment and adverse events evaluation

At baseline, angiomyolipomas were visualized and measured by abdominal computed tomography (CT) or magnetic resonance imaging (MRI), and the modality was kept consistent throughout the study for each patient. Up to four angiomyolipomas with maximum diameters ≥ 3.0 cm were identified as target lesions, and the sum of these diameters was obtained for each patient.

During the follow-up period, physical examination, blood routine, urine routine and radiographic evaluation of the kidneys were performed. Tumor response was assessed with Response Evaluation Criteria in Solid Tumors (RECIST version 1.1), while adverse events (AEs) were assessed according to the National Cancer Institute Common Terminology Criteria for Adverse Events (CTCAE version 4.0).

### Statistical analysis

Continuous variable, the decrease from baseline, was reported as mean ± standard deviation (M ± SD). Categorical variables were reported as frequency counts and percentages (%), including partial response (PR) and AEs. All statistical analyses were performed with SPSS software, version 19.0 (SPSS, Chicago, IL, USA). Student’s *t*-test was used to compare continuous variables, while Chi-square test, Fisher’s exact test and Yate’s continuity corrected chi-square test were used to compare categorical variables, as appropriate. All reported p values were 2-sided and p < 0.05 was considered statistically significant.

## Results

### Patient characteristics

A total of 124 TSC-AML patients from three clinical centers were reviewed. They were divided into sirolimus group (n = 33) and everolimus group (n = 91) according to the monotherapy they received. Baseline demographic characteristics were generally balanced between the two groups (Table 1). In general, patients in the everolimus group had relatively more severe renal phenotypes than patients in the sirolimus group, including more target AML lesions (≥ 3 cm) and larger size of target AML. Regarding to TSC-AML, 3–4 of target AML lesions (≥ 3 cm) were observed in 19 (58%) patients in the sirolimus group, while in 57 (63%) patients in the everolimus group. The median maximum diameters of target AML at baseline were 6.1 (3.5–16) cm and 8.1 (3.1–22) cm in the sirolimus group and the everolimus group, respectively. In addition, 36 (40%) patients in the everolimus group had received prior surgery/invasive procedure, and similarly, 15 (45%) patients in the sirolimus group had received such treatment.

**Table 1:** Baseline demographic and clinical characteristics

	Sirolimus n = 33 (%)	Everolimus n = 91 (%)
Age in years, median (range)	30 (19–59)	29 (18–52)
< 30 year	16 (48)	51 (56)
≥ 30 year	17 (52)	40 (44)
Sex		
Male	10 (30)	34 (37)
Female	23 (70)	57 (63)
LAM (female)	5 (22)	11 (19)
≥ 1 Skin lesion	31 (94)	89 (98)
SEGA	2 (6)	5 (5)
Maximum diameter of target AML		
Median (range, cm)	6.1 (3.5–16)	8.1 (3.1–22)
≥ 8 cm	12 (36)	48 (53)
≥ 4 cm and < 8 cm	16 (48)	37 (41)
≥ 3 cm and < 4 cm	5 (15)	6 (6)
Number of target AML lesions (≥ 3 cm)		
1–2	14 (42)	34 (37)
3–4	19 (58)	57 (63)
Surgery/invasive procedure	15 (45)	36 (40)
Renal embolisation	5 (15)	21 (23)
Partial nephrectomy	7 (21)	10 (11)
Nephrectomy	6 (18)	11 (12)

Descriptive statistics are frequencies and percentages or otherwise specified

*AML* angiomyolipoma,* LAM* lymphangioleiomyomatosis,* SEGA* subependymal giant cell astrocytoma

### Treatment efficacy

In the everolimus group, 3 patients dropped out at 6 months, 3 patients dropped out at 12 months and 4 patients dropped out at 24 months. The total drop-out rate was 11%. While the total drop-out rate was 15% in the sirolimus group, as 3 patients dropped out at 12 months and 2 patients dropped out at 24 months.

**Fig. 1:**
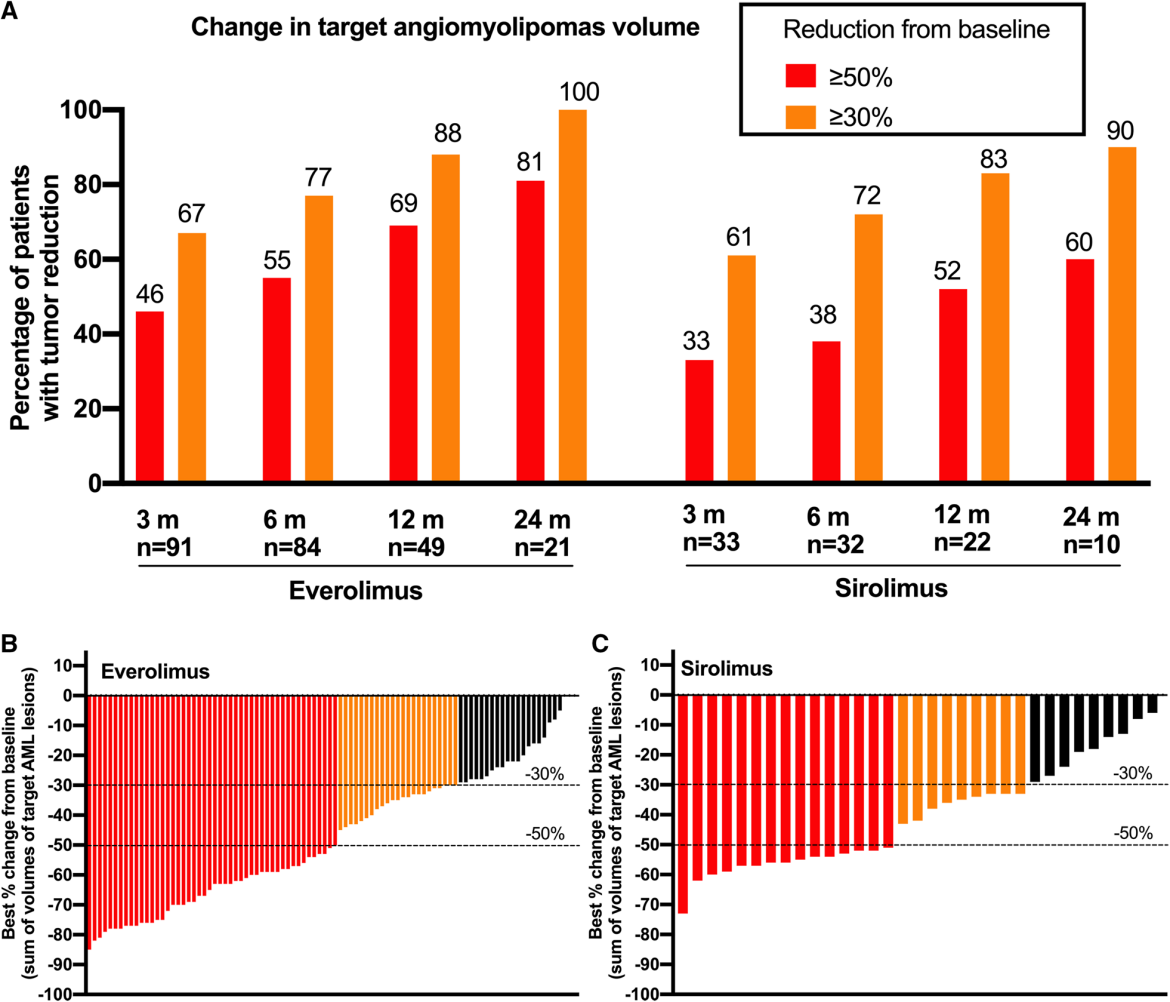
Change in target AML volume. **a** Proportions of patients treated with everolimus/sirolimus with a partial response or complete clinical response in target SML lesions per physician’s global assessment at 3, 6, 12 and 24 months. **b** Best percentage change from baseline in the sum of volumes of target AML lesions in everolimus patients, per central radiology review. Each bar represents one patient. **c** Best percentage change from baseline in the sum of volumes of target angiomyolipoma lesions in sirolimus patients, per central radiology review. Each bar represents one patient

**Fig. 2:**
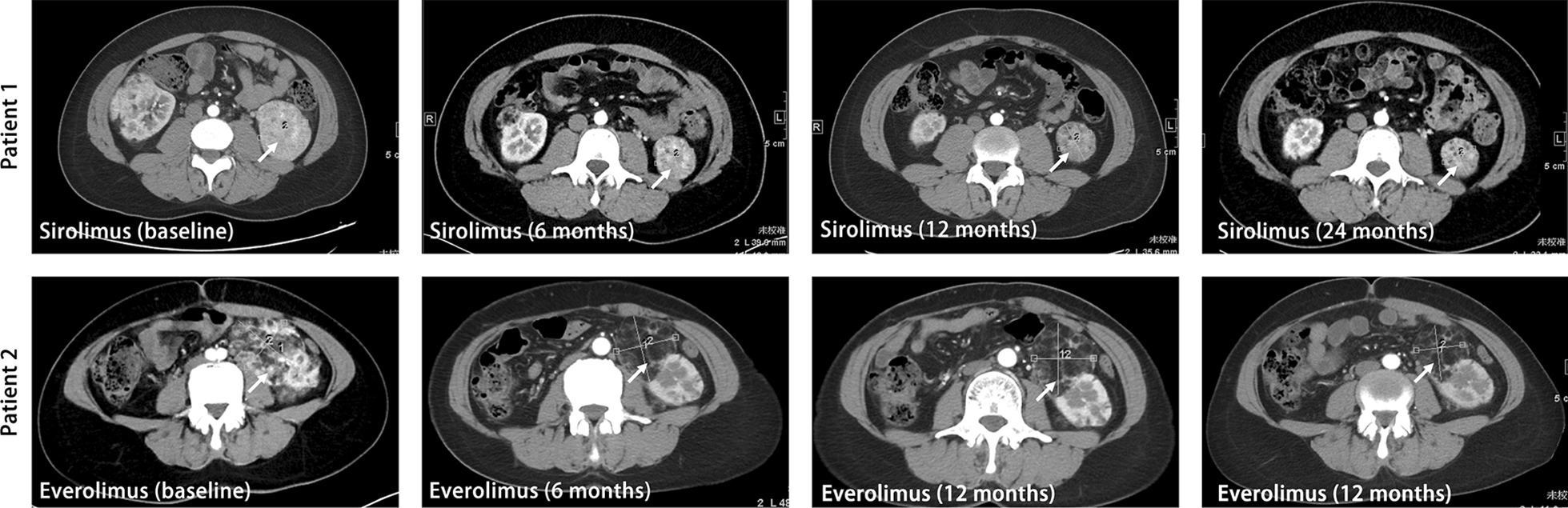
MRI imaging of two representative patients with TSC-AML in everolimus/sirolimus groups during follow-up

First, we compared the changes in target AML volume between these two groups. With the extension of duration, the number of patients who insist on medication in the two groups gradually decreased, and the percentage of patients with tumor reduction (≥ 30% and ≥ 50%) gradually increased. At all four follow-up points, the percentage of patients with tumor reduction (≥ 30% and ≥ 50%) in the everolimus group was higher than that in the sirolimus group (Fig. 2a). In general, everolimus surpassed sirolimus as it achieved better outcomes and demonstrated higher efficacy, as more patients in the everolimus group achieved more than 60% tumor volume reduction, even up to 85% tumor reduction. It’s worth noting that more than half of patients in the everolimus group achieved ≥ 50% reduction of target AML lesions from baseline. In contrast, less than half of the sirolimus patients achieved ≥ 50% reduction, and most of them fall within the range of 50–60% reduction. Moreover, regarding the patients with ≥ 30% tumor reduction after treatment, the percentage in the everolimus group was also higher than that in the sirolimus group (Fig. 1b, c).

Patient 1 and 2 were identified as representative cases in the everolimus group and the sirolimus group, since they presented a remarkable reduction of target AML volume, which could be visualized on MRI (Fig. 2).

Moreover, we set the decrease from baseline and response rate as variables to compare efficacy of the two agents (Table 2). Almost all patients showed a great response to mTOR inhibitors treatment. With the extension of treatment duration, the decrease from baseline became pronounced, the response rate gradually increased as well. After 3 months of treatment, the sirolimus group and the everolimus group showed similar response rates (33% vs. 46%) and similar decrease from baseline (35.4 ± 16.2% vs. 42.0 ± 17.6). Intriguingly, after prolonged treatment, everolimus showed higher efficacy than sirolimus on decrease from baseline (6 months: 48.5 ± 20.6% vs. 38.1 ± 17.2%, P = 0.01; 12 months: 56.7 ± 21.2% vs. 45.1 ± 13.9%, P = 0.02). However, no statistical difference was found in the response rates between the two groups at these two follow-up points (6 months: 38% vs. 55%; 12 months: 52% vs. 69%). At last follow-up point of 24 months, the response rates (60% vs. 81%) and decrease from baseline (52.5 ± 14.1% vs. 62.5 ± 15.3%) didn’t exhibit any significant differences between the sirolimus group and the everolimus group.

**Table 2:** Treatment efficacy

	Sirolimus	Everolimus	*P*-Value
3 months			
Decrease from baseline (mean ± SD, %)	35.4 ± 16.2	42.0 ± 17.6	0.06
No. PR /Total No. patients (%)	11/33 (33)	42/91 (46)	0.20
6 months			
Decrease from baseline (mean ± SD, %)	38.1 ± 17.2	48.5 ± 20.6	0.01
No. PR /Total No. patients (%)	12/32 (38)	46/84 (55)	0.10
12 months			
Decrease from baseline (mean ± SD, %)	45.1 ± 13.9	56.7 ± 21.2	0.02
No. PR /Total No. patients (%)	12/23 (52)	34/49 (69)	0.16
24 months			
Decrease from baseline (mean ± SD, %)	52.5 ± 14.1	62.5 ± 15.3	0.09
No. PR /Total No. patients (%)	6/10 (60)	17/21 (81)	0.38

Target angiomyolipomas in the kidney in each patient were visualized by CT or MRI at baseline and at 3, 6, 12, 18 and 24 months and the longest diameter of each angiomyolipoma was measured

*PR* partial response, defined as a reduction in angiomyolipoma volume (sum of volumes of all target angiomyolipomas identified at baseline) of 50% or more relative to baseline and absence of angiomyolipoma progression

### Adverse events

The Adverse events of two agents during treatment were shown in Table 3. A variety of common AEs have been reported in both the sirolimus group and the everolimus group without statistical differences, including oral mucositis (91% vs. 98%), irregular menstruation (30% vs. 44%), hypertriglyceridemia (33% vs. 33%), upper respiratory infection (33% vs. 24%), and rash acneiform (18% vs. 18%). Grade 3 or 4 AEs occurred in 11 (33%) and 26 (29%) patients in the sirolimus group and the everolimus group, respectively. No unexpected AEs were reported. No patient refused treatment or exited the cohort due to AEs, and no mortality was reported during medication.

**Table 3:** Everolimus/Sirolimus-related adverse events

	Sirolimus n = 33 (%)		Everolimus n = 91 (%)		*P*-value All grades
	All grades	Grade 3–4	All grades	Grade 3–4	
Gastrointestinal					
Mucositis oral	30 (91)	4 (12)	89 (98)	11 (12)	0.23
Diarrhea	4 (12)	0	5 (5)	0	0.39
Vomiting	0	0	3 (3)	0	0.69
Constipation	2 (6)	0	1 (1)	0	0.35
Gynaecological					
Irregular menstruation (female)	7 (30)	3 (13)	25 (44)	9 (15)	0.27
Metabolic					
Hypertriglyceridemia	11 (33)	0	30 (33)	0	0.97
Cholesterol high	4 (12)	0	11 (12)	0	0.76
Proteinuria	2 (6)	0	8 (9)	0	0.90
ALP increased	4 (12)	0	8 (9)	0	0.83
GGT increased	2 (6)	0	5 (5)	0	0.75
Hypophosphatemia	0	0	3 (3)	0	0.69
Infection					
Upper respiratory infection	11 (33)	0	22 (24)	0	0.31
Pneumonitis	4 (12)	2 (6)	5 (6)	3 (3)	0.39
Urinary tract infection	4 (12)	0	5 (6)	0	0.39
Dermatology					
Rash acneiform	6 (18)	0	16 (18)	3 (3)	0.94
Constitutional symptoms					
Abdominal pain	4 (12)	0	11 (12)	0	0.76
Headache	4 (12)	0	3 (3)	0	0.15
Malaise	2 (6)	0	3 (3)	0	0.86
Soft tissues					
Peripheral oedema	4 (12)	2 (6)	3 (3)	0	0.15
Blood					
Neutrophil count decreased	2 (6)	0	3 (3)	0	0.86
Lymphocyte count decreased	0	0	3 (3)	0	0.69
Anemia	2 (6)	0	1 (1)	0	0.35

Everolimus/Sirolimus-related adverse reactions were those adverse events that were considered to be possibly or definitely related to everolimus/sirolimus

## Discussion

Before everolimus was included in Chinese medical insurance, the relatively low economic expenditure and wide application scope of sirolimus had prompted its option over everolimus for many TSC-AML patients under low economic conditions, though everolimus exhibited many advantages in pharmacokinetics. However, with the price decreasing and the indication widening, more and more people choose everolimus for TSC-AML treatment. This may explain the result that numbers of participants receiving sirolimus/everolimus were not equal. We thus wondered whether sirolimus can achieve considerable curative effect and become an alternative choice of everolimus for TSC-AML patients who can’t afford the cost of everolimus. To the best of our knowledge, this Chinese multi-institutional retrospective study is the first to compare the efficacy of everolimus and sirolimus for the treatment of TSC-AML patients.

The changes in target AML volume exploratory study showed a considerable improvement in the volume of target AML with the mTOR inhibitors treatment, consistent with previous studies [14, 15]. In the everolimus group, more than half of patients had ≥ 50% reduction from baseline of target AML lesions,and approximately 80% patients had ≥ 30% reduction. This is basically in agreement with the results from a double-blind, placebo-controlled, phase 3 trial of everolimus, that 55% of everolimus patients had ≥ 50% reduction from baseline in sum of volumes of target angiomyolipoma lesions and 80% of everolimus patients had ≥ 30% reduction [6]. In terms of the percentage of patients with tumor reduction (≥ 30% and ≥ 50%), everolimus surpassed sirolimus as it achieved better outcomes and demonstrated higher efficacy. Further statistical analysis also demonstrated both agents could achieve high response rate and significant tumor reduction. And with the extension of medication time, the efficacy was more and more obvious. This confirms that everolimus and sirolimus, as mTOR inhibitors, do have definite efficacy in the treatment of TSC-AML as both of them can significantly reduce TSC-AML volume. Importantly, everolimus outperformed sirolimus in decreasing the target AML volume after 6 months and 12 months of medication although there was no statistical difference in the response rate between the two groups. In general, everolimus has more obvious advantages than sirolimus in the TSC-AML treatment in our study. Of interest, after 24 months, everolimus didn’t lead to more significant reduction in tumor volume than sirolimus as expected, which may be attributed to the limited number of patients who received medication for 24 months. Generally speaking, patients are more likely to seek alternative treatment if the current therapy fails. Thus, only 31 patients persisted with medication for 24 months, including 21 in the everolimus group and 10 in the sirolimus group. The lower sample size at follow-up after 24 months reduces the reliability of the long-term efficacy comparison between everolimus and sirolimus. Studies with larger sample size and sustained follow-up are needed to compare the long-term efficacy of the two agents. In addition, A previous study found a trend toward larger volume in TSC-AML after cessation of mTOR inhibitors therapy, which was also reported in our previous work [14, 16]. Thus, it is important to assess whether continuous mTOR inhibitors is necessary to maintain the reduction in the total volume of target AML.

The safety evaluations showed that everolimus and sirolimus had a similar and acceptable safety profile with no statistical difference. The types and incidence of AEs were consistent with other cohort studies, including oral mucositis, irregular menstruation, hypertriglyceridemia, upper respiratory infection, and rash acneiform [5, 15]. Among those AEs, oral mucositis stood as the most common type (91% in the sirolimus group; 98% in the everolimus group). AEs of grade 3 or 4 only occurred in a small part of patients, which further confirms the safety of mTOR inhibitors used in TSC-AML treatment. However, Bissler JJ has reported serious AEs of sirolimus, which was attributed to patients’ poor physical conditions before medication [14]. It reminds us that we should be especially cautious in employing sirolimus to these patients with chronic diseases. Similar to previous studies, oral mucositis with high incidence was observed in our study, suggesting the need for appropriate monitoring and management [5, 15]. Theoretically, mTOR inhibitors can promote the release of keratinocyte cytokines, directly cause epithelial injury, and eventually lead to stomatitis [19]. Of course, the details may be more complicated and require further exploration and verification at the molecular and animal levels. In order to avoid oral mucositis, patients should be instructed to maintain good oral hygiene by the frequent mouth wash using non-alcoholic reagents like 0.9% normal saline [20]. To alleviate oral mucositis, topical treatment with sucralfate or oral rinses with dexamethasone are recommended [20, 21]. In addition to oral mucositis, irregular menstruation also occurs frequently in female patients, accounting for about 30% in the sirolimus group and 44% in the everolimus group, which has been frequently reported before [6, 8, 15]. Disturbed hormone levels and ovarian cysts associated with mTOR inhibitor therapy have also been described [22–24], although the concrete mechanism was unknown. As reported in a two-year trial in China, more than 90% of women experienced amenorrhea subsequent to the mTOR inhibitor therapy [16]. Clinicians should pay attention to the effects of mTOR inhibitors on menstruation in female patients of childbearing age.

There were several limitations in our study. Firstly, the number of participants in the everolimus group was different from that in the sirolimus group, which resulted in a certain bias. Meanwhile, the sample size enrolled in our cohort is not big enough, especially at the follow-up point of 24 months after medication. Future studies focusing on this filed should employ independent cohorts with larger sample sizes to investigate the efficacy and safety of everolimus and sirolimus in patients with TSC-AML. Secondly, the monitoring of blood drug concentration, which can make the results more convincing and ensure the safety of drug use, it was not performed in our study. Finally, a small number of patients in the sirolimus group chose domestic sirolimus due to economic considerations. We assumed that the efficacy of domestic sirolimus is equivalent to that of imported sirolimus, but whether their efficacies are consistent remains unknown.

## Conclusions

In view of the efficacy and safety associated with reductions in target AML in TSC patients, both everolimus and sirolimus are good therapeutic options. Although sirolimus requires less economic expenditure, everolimus demonstrates greater therapeutic efficacy in reducing TSC-AML volume. Therefore, we recommend everolimus as first-line treatment, especially for patients with more severe TSC- AML.

## Data Availability

Please contact author (Yi Cai, cai-yi@csu.edu.cn) for data requests.
